# Anti-Cancerous Potential of Polysaccharides Derived from Wheat Cell Culture

**DOI:** 10.3390/pharmaceutics14051100

**Published:** 2022-05-20

**Authors:** Alima Murtazina, Gloria Ruiz Alcala, Yaiza Jimenez-Martinez, Juan Antonio Marchal, Anel Tarabayeva, Elmira Bitanova, Gordon McDougall, Nazira Bishimbayeva, Houria Boulaiz

**Affiliations:** 1 Biopathology and Regenerative Medicine Institute (IBIMER), Centre for Biomedical Research, University of Granada, 18100 Granada, Spain or murtazina@correo.ugr.es (A.M.); glorual@correo.ugr.es (G.R.A.); yaijmartinez@correo.ugr.es (Y.J.-M.); jmarchal@ugr.es (J.A.M.); 2 Department of General Immunology, Faculty of Medicine, Asfendyarov Kazakh National Medical University, Almaty A35B8H9, Kazakhstan; tarabaeva.a@kaznmu.kz (A.T.); bitanova.e@kaznmu.kz (E.B.); 3 Research Center “Bioscience Technologies”, Almaty A15G7B0, Kazakhstan; 4 Instituto de Investigación Biosanitaria ibs. GRANADA, University Hospitals of Granada, University of Granada, 18012 Granada, Spain; 5 Department of Human Anatomy and Embryology, Faculty of Medicine, University of Granada, 18012 Granada, Spain; 6 Research Unit “Modeling Nature” (MNat), University of Granada, 18016 Granada, Spain; 7 Plant Biochemistry and Food Quality Group, Environmental and Biochemical Sciences Department, The James Hutton Institute, Invergowrie, Dundee DD2 5DA, UK; gordon.mcdougall@hutton.ac.uk; 8 Department of Biotechnology, Faculty of Biology and Biotechnology, Al-Farabi Kazakh National University, Almaty A15E3B4, Kazakhstan

**Keywords:** wheat cell culture, plant polysaccharides, colon cancer cells, inhibition of proliferation, electron microscopy, apoptosis, cytochrome c, differentiation

## Abstract

There is a global need to discover effective anti-cancerous compounds from natural sources. Cultivated wheat cells can be a valuable source of non-toxic or low toxic plant-derived polysaccharides. In this study, we evaluated the anti-cancer ability of seven fractions of wheat cell culture polysaccharides (WCCPSs) in the HCT-116 colon cancer cell line. Almost all (6/7) fractions had an inhibitory effect on the proliferation of colon cancer cells, and two fractions (A-b and A-f) had considerable therapeutic indexes. The WCCPS fractions induced cell cycle arrest in the G1 phase and induced different rates of apoptosis (≤48%). Transmission and scanning electron microscopy revealed that WCCPS fractions caused apoptotic changes in the nucleus and cytoplasm, including damage to mitochondria and external morphological signs of apoptosis. In addition, the WCCPSs induced an increase in the levels of Bax, cytochrome c, and caspases 8 and 3, indicating that cell death progressed through intrinsic and extrinsic pathways of apoptosis. Furthermore, some fractions caused a significant decrease of c-Myc, b-catenin, NFkB2, and HCAM (CD 44) levels, indicating enhanced cell differentiation. Thus, for the first time, our results provide a proof of concept of the anti-cancer capacity of WCCPS fractions in colorectal cancer.

## 1. Introduction

Despite recent developments in prevention and treatment, cancer causes millions of annual deaths and carries a huge worldwide economic burden [1]. In 2020, cancer accounted for 10.0 million deaths worldwide, including colorectal cancer (CRC) which led to 915,000 deaths [2]. On a global scale, between one and two million new cases of CRC are diagnosed every year, making it the third most common cancer and the fourth most common cause of cancer-related death [3,4,5]. Although recent achievements in cancer treatment are promising, there is still a constant need to search for new compounds that could fight cancer with fewer side effects on non-cancerous cells. Compounds extracted from natural sources have been of special interest in recent decades as a source of effective cancer treatment and prevention [6,7]. It is therefore urgent to search for active compounds from natural sources that could be effective in the prevention and treatment of this pathology.

In the past few years, there has been an increased interest in the exploration of new compounds derived from renewable natural sources for safe and effective cancer treatment and prevention. Polysaccharides (PSs) are related to the class of natural substances that have been obtained from microbes, fungi, algae, and plants. It has been shown that they have immunomodulating antioxidant properties and antitumor effects both in vitro and in vivo [7,8,9]. The biological activity of natural PSs has been shown in preclinical studies to be able to diminish tumor growth and prolong survival by immune stimulation, apoptosis, and cell cycle arrest [10].

In the past two decades, there has been a tendency to search for new polysaccharides with anticancer properties that cause minimal harm to the host cell. In this regard, polysaccharides isolated from plants have often been considered preferable to other sources, because they are less toxic and cause fewer side effects [11]. Moreover, plant polysaccharides are complex macromolecules with wide structural and functional variability [12,13], which makes them powerful molecules with many biological effects. Polysaccharides derived from a variety of plants (apple, ginseng, aloe, and many others) have already been proved to have anti-tumor, immunomodulating, and antioxidant properties [14,15].

The antioxidant activity of polysaccharides has been shown to have a protective effect against cellular oxidation damage, minimizing the hazard caused by reactive oxygen species. At the same time, it is known that many chronic diseases (including cancer, diabetes, cardiovascular diseases, etc.) are associated with oxidative stress and polysaccharides can act as considerable free radical scavengers [16,17]. Thus, the huge magnitude of the beneficial effects of polysaccharides makes them highly valuable for preventing and treating cancer and many other chronic disorders.

Among plant PSs, medicinal herbs have been investigated more frequently, and have been used in cancer adjuvant therapy. Some of the PSs from mushrooms and medicinal plants have gone on to become pharmaceutical products such as lentinan, schizophyllan/sizofiran/sonifilan, Krestin, GanoPoly, astragalan, GCS-100, and PectaSol, with low toxicity and potent antitumor activity. In Japan, China, and Korea, bioactive PSs have already been introduced as an adjuvant alongside standard radio- and chemotherapy in cancer treatment [10]. Moreover, clinical trials in the USA showed the beneficial effect of PSs when added to adjuvant therapy for different kinds of cancer [18,19].

The chemical structure and functional characteristics were investigated for cereal polysaccharides, which were suggested because of their use in the food, cosmetics, and medicine industries [20,21]. There is evidence of anti-cancer and immunomodulating activity that was reported for rice bran polysaccharides [22]. Recently, the antitumor effect of wheat bran polysaccharides has also been revealed [23]. However, there is no study on the biological effect of PSs obtained from the cell culture of cereals.

It has been shown that plant callus tissues can be a source of valuable polysaccharides [24,25]. Cultivating plant cells is a biotechnological approach that enables different chemical compounds to be obtained with a number of advantages: no organismic control, an absence of changes in climatic conditions, homogeneity, and the possibility for optimization, standardization, and obtaining a higher output [26]. In vitro plant tissue cultivation makes it possible to target production of polysaccharides that are of special value [27]. Moreover, plant cell culture allows the production and regulation of the output of phytochemicals with biotransformation, which contributes to the acquisition of natural substances with novel activities [28]. Furthermore, plant cell culture technologies surpass the benefit of agricultural production of commercially important compounds on an economic scale. For example, in the production of paclitaxel, approved by the FDA, it is produced in 75 m^3^ bioreactors with an output of 500 kg per year. However, to achieve a high output of cell culture, it is crucial to screen for productive plant cell lines and attempt to optimize culture growth conditions [29].

Our previous research enabled us to identify bioactive polysaccharides in cereal cell suspension cultures [30]. We explored the chemical composition and physical properties of these wheat PSs, which were found to be represented by arabinogalactans and supposed to be represented by xyloglucans, arabinoxylans, and glucans, according to their monosaccharide composition [31,32]. In addition, we showed that monosaccharide composition changed significantly depending on the cultivation media [33]. It is known from the literature that these types of polysaccharides are considered applicable to medicine, including for cancer treatment [10]. Thus, evidence suggests that arabinogalactans [34], beta-glucans [35], and arabinoxylans [36] have the ability to suppress proliferation of cancer cells in vitro [36].

Based on these observations, in this study we analyzed the anti-cancerous effect of wheat cell culture polysaccharides (WCCPSs) on CRC cells. To achieve this, colon cancer cells were treated with seven fractions of WCCPSs to evaluate their cytotoxic impact. Moreover, morphological change, cell cycle, and apoptosis-related proteins were analyzed to assess the molecular mechanism of WCCPS-induced cell death/inhibition of proliferation in cancer cells. Furthermore, we determined the value of the therapeutic index (TI) of WPPSS fractions using normal colon cells, thus exploring new and more economical and sustainable strategies for CRC treatment.

## 2. Materials and Methods

### 2.1. Plant Cell Culture, Polysaccharides Purification and Separation

Cell suspension cultures of soft spring wheat *Triticum aestivum* of the Kazakhstanskaya 10 variety (provided by Kazakh Research Institute of Agriculture and Plant Growing, Almaty, Kazakhstan) were grown in two types of Murashige and Skoog (MS) [37] liquid medium (Sigma-Aldrich, St. Louis, MO, USA) as we described before [33] with the addition of phytohormones which are commonly used in plant biotechnology to regulate morphogenesis and biosynthesis of secondary bioactive metabolites in vitro [38,39,40,41]: 5.0 mg/L 2,4-D—2,4-dichlorophenoxyacetic acid (Sigma-Aldrich, St. Louis, MO, USA) and 1.0 mg/L ABA—abscisic acid (Sigma-Aldrich, St. Louis, MO, USA).

For obtaining the cell suspension culture, 200 mg of wheat callus tissues, grown on solid medium, was placed in 30 mL of liquid culture medium in laboratory flasks (Sigma-Aldrich, St. Louis, MO, USA) and cultivated in an ES-20 Incubator Shaker (BioSan, Riga, Latvia) at 140 rpm, 26 ± 2 °C for a 16 h photoperiod with light intensity of 10 μmol m^−2^ s^−1^ for one, three, and six weeks. The resulting cell culture media were collected by filtration using Whatman qualitative filter paper (Merck, Darmstadt, Germany) then concentrated in a rotor evaporator (IKA, RV 3 V, Staufen, Germany) and used for polysaccharides extraction.

Polysaccharide Preparation and Purification: Polysaccharides were precipitated from the media by adjusting to 70% (*v*/*v*) ethanol (Sigma-Aldrich, St. Louis, MO, USA) and incubating at 4 °C for at least 4 h. The pelleted polysaccharides were collected by centrifugation at 10,000 rpm for 10 min at 8 °C using refrigerated centrifuge 5810R (Eppendorf, Gamburg, Germany). The enrichment in the total amount of sugar was confirmed using the Dubois method [42] using a SmartSpec spectrophotometer (Biorad, Hercules, CA, USA). The precipitated PS samples were resuspended in ultrapure water (<18 MΩ, Elga water systems, High Wycombe, UK), then freeze-dried (Martin Christ ALPHA 1-2 LD plus, Osterode am Harz, Germany).

Separation. The total polysaccharide (PS) sample T-010 was separated into bound (acidic) and unbound fractions using ion-exchange chromatography on DEAE-Sepharose (Sigma-Aldrich, St. Louis, MO, USA) as previously reported [43]. In brief, the PS samples were dissolved in 10 mL of 100 mM Tris HCl buffer pH 8.0 and then applied to a column of DEAE-Sepharose (100 mL) which had been equilibrated in the same buffer. The unbound material was eluted using two-bed volumes of the same buffer then the bound material was eluted using 100 mM Tris HCl containing 0.5 M NaCl. The sugar content was confirmed using the Dubois method [42] and the unbound and bound PS samples were recovered by precipitation in 70% ethanol, as above. The precipitated bound and unbound PS samples were resuspended in ultrapure water and then freeze-dried.

### 2.2. Characterization of Polysaccharide Fractions, Monosaccharide Content Determination

**Figure 1:**
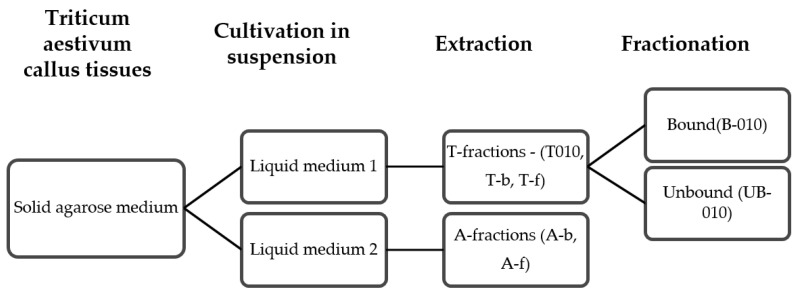
Scheme of PS fractions obtainment.

In total, seven PS fractions were obtained and used for the study of the effect on CRC cells and their mechanism of action. These fractions differed from each other in terms of the nutrient media for wheat cell cultivation, cultivation time, and fractionation (Figure 1).

Wheat cells were grown in media 1 with 5.0 mg/L 2,4-D and media 2 with 1.0 mg/L ABA [33]. The WCCPS fractions that we used were obtained from short-term (7 days), medium-term (21 days) and long-term (42 days) cultivation, because we observed different composition of PSs depending on the cultivation time. Cells grown in the short-term provided the T-010 and A-b fractions, the medium-term growth provided the T-b fraction, and the long-term growth provided the T-f and A-f fractions. The T-010 fraction was fractionated by ion-exchange chromatography. As a result, we obtained 5 unfractionated fractions, and two fractions obtained by ion-exchange chromatography: the acidic and neutral-basic fractions (B-010 and UB-010, respectively).

The monosaccharide composition of the polysaccharides in certain fractions was investigated by using acid hydrolysis followed by high-performance anion exchange chromatography (HPAEC) [44]. Briefly, samples containing 10 mg of each fraction were hydrolyzed in triplicate using 1 mL of 2 M trifluoroacetic acid (Sigma-Aldrich, St. Louis, MO, USA) at 120 °C for 2 h. After centrifugation (10,500× *g*, 5 min), the samples were dried in a Speed-Vac (Genevac Ltd. miVac Duo, Ipswich, UK) then resuspended in ultrapure water. After suitable dilution, their monosaccharide composition was assessed against standard curves of relevant monosaccharides separated on a CarboPAC PA20 column and detected using pulsed amperometric detection (Dionex ICS-5000 system, Thermo Fisher Scientific Inc., Waltham, MA, USA).

All polysaccharide fractions were dissolved in ultrapure water for stock solutions. For each experiment, the stock solutions were further diluted in DMEM medium to obtain the desired concentrations.

### 2.3. Cell Lines

The HCT-116 colon cancer cell line and CDD-118CO normal colon cell line were obtained from American Type Culture Collection (ATCC, Manassas, VA, USA) and maintained in Dulbecco’s Modified Eagle Medium (DMEM; Sigma-Aldrich, St. Louis, MO, USA) supplemented with 10% fetal bovine serum (FBS). All the cell lines were authenticated using short-tandem repeat profiling and were passaged for less than 6 months, and routinely assayed for mycoplasma contamination.

### 2.4. In Vitro Cytotoxicity Assays

The effect of PS fractions on cell viability was assessed using the Thiazolyl Blue Tetrazolium Bromide (TBTB) (Sigma, St. Louis, MO, USA) colorimetric assay (MTT). Briefly, cells (2.5 × 10^3^ cells/well) were seeded on 96-well plates, incubated for 24 h and then treated with different PS concentrations. Three days later, the wells were aspirated and treated by TBTB for 3 h, followed by dimethyl sulfoxide (DMSO) dissolution (≥99.5%) (Sigma-Aldrich, Saint-Quentin Fallavier, France). Cells were processed, as previously, using a Titertek Multiscan apparatus (Flow, Irvine, CA, USA) at 570 nm. We evaluated the linearity of the assay by calculating the number of cells in each well before every cell growth experiment. The 50% inhibition concentration (IC_50_) values were calculated from semi-logarithmic dose-response curves by linear interpolation [45]. For the non-monotonic response fraction, we considered the lowest concentration of around 50% of inhibition of proliferation as IC_50_. All the experiments were plated in triplicate wells and were carried out three times.

### 2.5. Apoptosis and Cell Cycle Assays

The cell cycle phases (G_0_/G_1_, S, or G_2_/M) were characterized by differences in cellular DNA content. As fluorescent dye propidium iodide (PI) (Sigma, St. Louis, MO, USA) strongly bound with DNA at a ratio of 1:1, DNA contents of the cell cycle phases had varied PI fluorescent intensities. HCT-116 cell lines were seeded in 6-well plates (200 × 10^3^ cells per well) and incubated for 48 h. They were then treated for 48 h by PS fractions in different concentrations determined by doubled IC_50_ levels. Cells in monolayer culture were harvested, washed twice with PBS, and fixed in ice-cold 70% ethanol at 4 °C. The cell pellets were washed twice in PBS and resuspended in a DNA extraction solution (PH = 7.8) of acetic acid (0.1 M) and sodium phosphate dibasic anhydrous (0.2 M) in PBS, incubated for 15 min at 37 °C. The cells were pelleted and washed once again with PBS and resuspended in PI/RNase/PBS (100 mg/mL PI, 40 mg/mL RNase, BD, Biosciences, Franklin Lakes, NJ, USA) solution in the dark for 30 min at 37 °C [46]. The percentage of cells in the subG_1_, G_0_/G_1_, S, and G_2_/M phases was determined by a FACS Calibur flow cytometer (BD, Biosciences, Franklin Lakes, NJ, USA).

### 2.6. Electron Microscopy

The HCT-116 cell line was seeded in 6-well plates (200 × 10^3^ cells per well) and incubated for 48 h. The cells were then treated for 48 h with the PS fractions in concentrations that varied according to double the IC_50_ levels. The cells were washed in ice-cold PBS, fixed and prepared for transmission electron microscopy (TEM) according to standard protocol (Libra 120 PLUS, Carl Zeiss SMT, Oberkochen, Germany) [47]. The HCT-116 cell line was seeded in 24-well plates (100 × 10^3^ cells per well) for scanning electron microscopy (SEM), after 48 h they were treated for 48 h using the same concentrations of PS. The cells were fixed, and samples were prepared for SEM (Hitachi S-800, Hitachi, Tokyo, Japan) as described in [48].

### 2.7. Western Blotting

The HCT-116 colon cancer cells were seeded on 6-well plates in DMEM medium. After 48 h of treatment, the medium was removed from the cells, and the cells were centrifugated at 1500 rpm, washed twice with PBS, and then lysed in RIPA lysis buffer (Santa Cruz Biotechnology, Dallas, TX, USA). Immunoblotting on whole cell lysates was performed following routine protocols [49]. The following primary antibodies were used for protein detection: caspase 3, dilution 1:1000 (Cell Signaling, Beverly, MA, USA); caspase 8, dilution 1:500 (caspase-8 (8CSP03): sc-56070, Santa Cruz Biotechnology, Dallas, TX, USA); bax, dilution 1:500 (bax (B-9): sc-7480, Santa Cruz Biotechnology, Dallas, TX, USA); cytochrome-c, dilution 1:500 (cytochrome c (7H8): sc-13560, Santa Cruz Biotechnology, Dallas, TX, USA), β-actin, dilution 1:15,000 (A2228, Sigma-Aldrich, St. Louis, MO, USA), c-Myc, dilution 1:100, (c-Myc (9E10): sc-40,Santa Cruz Biotechnology, Dallas, TX, USA), beta-catenin, dilution 1:100 (p-beta-catenin (1B11): sc-57533, Santa Cruz Biotechnology, Dallas, TX, USA); NF-kB2, dilution 1:1000, (NF-kB2 p100/p52, 4882, Cell signaling technology, Beverly, MA, USA); CD44, dilution 1:200, (HCAM (DF1485): sc-7297, Santa Cruz Biotechnology, Dallas, TX, USA). Secondary antibodies used included anti-rabbit IgG peroxidase conjugate (A0545, Sigma-Aldrich, St. Louis, MO, USA) and anti-mouse IgG peroxidase conjugate (A9044, Sigma-Aldrich, St. Louis, MO, USA). The protein–antibody complexes were made visible using enhanced chemiluminescence (ECL, Bonus, Amersham, Little Chalfont, UK) with the IMAGE READER LAS-4000 program in a LAS-4000 imaging system. The interpretation of the intensity of the signal was done using the Image J program. The values of each band were normalized by dividing them by the value of their β-actin, and they were relativized with respect to the control sample to which the value 1 was assigned. In total, three membranes were implemented for protein detection. cMyc (67 kDa), Cyt C (14 kDa), and NFkB p100 were determined in one membrane. Another membrane was used to detect Bax (23 kDa), Caspase 3 (35 kDa), Caspase 8 (55 kDa), and CD44 (90–95 kDa). Beta-catenin was revealed on a separate membrane. All the experiments were carried out at least three times.

### 2.8. Statistical Analysis

All experiments were done in at least 3 replicates. TEM and SEM cells were seeded in four replicates. The data are expressed as means ± standard deviation (SD). The Student’s *t*-test was used to determine the statistical significance of differences between the two groups. A *p*-value of 0.05 or less was considered to be statistically significant. The GraphPad Prism 8 program was used to produce the figures. The 50% inhibition concentration (IC_50_) values were calculated using different approaches because of non-monotonic concentration-response or non-achieved IC_50_. To assess statistically significant changes in concentrations, we used linear interpolation from semi-logarithmic dose-response curves (A-b, T-f, T-b). For fractions that had not achieved IC_50,_ we considered the highest concentration that caused maximum inhibition (for T-010 and UB-010 fractions). For the A-f fraction, no statistically significant differences were observed across concentrations, and no logarithmic linear interpolation could be applied, therefore we used minimal concentration with an IC_50_ value.

## 3. Results

### 3.1. Monosaccharide Composition of WCCPSs

The relative monosaccharide composition of the fractions determined by HPAEC is shown in Table 1 and in the Supplementary Data (Figure S1) and was used to suggest the nature of their polysaccharide component. In brief, the presence of glucose suggested the presence of glucans, the presence of arabinose and galactose, arabinogalactans, and the presence of glucuronic acid and xylose, glucuronoarabinoxylans. However, the presence of xylose and glucose could also indicate the presence of xyloglucans.

The monosaccharide composition of T-010 sample was composed of glucose (74%), arabinose (9%), galactose (10%), xylose (7%) had the following ratio Ara:Gal:Xyl:Glc:GlcUA:GalUA:Man = 9:10:7:74:0:0:0 (Table 1). The B-010 fraction comprised arabinose (33%), galactose (21%), xylose (18%), glucose (3%), and glucuronic acid (25%), and had the following ratio Ara:Gal:Xyl:Glc:GlcUA:GalUA:Man = 33:21:18:3:25:0:0. The UB-010 fraction consisted of arabinose (8%), galactose (6%), xylose (10%), glucose (76%), and had the following ratio Ara:Gal:Xyl:Glc:GlcUA:GalUA:Man = 8:6:10:76:0:0:0. The monosaccharide composition of the A-b sample was glucose (92%), arabinose (1.4%), galactose (0.7%), xylose (1.9%), glucuronic acid (0.4%), galacturonic acid (0.6%), and mannose (3.0%) and had the following ratio Ara:Gal:Xyl:Glc:GlcUA:GalUA:Man = 1.4:0.7:1.9:92:0.4:0.6:3.0 (Table 1). The monosaccharide content of the A-f sample showed a composition of glucose (83%), arabinose (7%), galactose (3%), xylose (5%), glucuronic acid (1%), and had the following ratio 7:3:5:83:1:0:0. The composition of the T-b fraction showed a content of glucose (57%), arabinose (8%), galactose (15%), xylose (14%), glucuronic acid (2%), galacturonic acid (0%), and mannose (4.0%) and had the following ratio Ara:Gal:Xyl:Glc:GlcUA:GalUA:Man = 8:15:14:57:2:0:4.0. The monosaccharide composition of the T-f sample consisted of glucose (58%), arabinose (16%), galactose (10%), xylose (14%), glucuronic acid (2%), galacturonic acid (0.6%), and mannose (3.0%) and had the following ratio Ara:Gal:Xyl:Glc:GlcUA:GalUA:Man = 16:10:14:58:2:0:0.

### 3.2. Wheat Cell Culture Polysaccharides Have an Antiproliferative and Selective Inhibitory Effect on Colon Cancer Cell Lines

**Figure 2:**
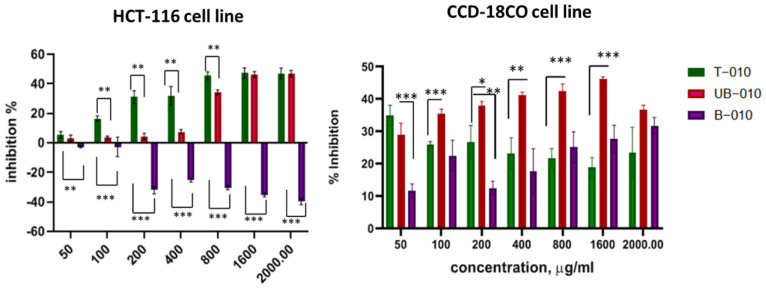
Inhibition of HCT-116 colon cancer cell line and CCD118 CO normal colon cell line proliferation (in %) under the influence of different concentrations of T−010, B−010, and UB−010 fractions. Fractionated PSs (T−010—total fraction, B−010—bound fraction, UB−010—unbound fraction). (*** *p*  <  0.001, ** *p*  <  0.01 and * *p*  <  0.05 versus control).

The cell viability assay revealed that WCCPSs inhibited proliferation of HCT-116 colon cancer cells at different doses. The T-010 fraction inhibited the proliferation of cancer cells to 47% ± 3.47 at a concentration of 1600 µg/mL. Fractionated samples (B-010, UB-010), obtained from the T-010 sample by ion exchange chromatography, had a differential effect (Figure 2). The unbound fraction (UB-010) had an antiproliferative effect on HCT-116 cells with a maximal inhibition of 46.2% ± 2.0 at 1600 µg/mL, whereas the bound fraction (B-010) stimulated cell proliferation in vitro up to 40.0% ± 2.2. Furthermore, the 3 fractions inhibited the proliferation of normal cells, with the UB fraction being the most toxic at low concentrations (Figure 2).

Taking these results into account, we excluded the B-010 fraction from further investigation as it stimulated the proliferation of tumor cells.

**Figure 3:**
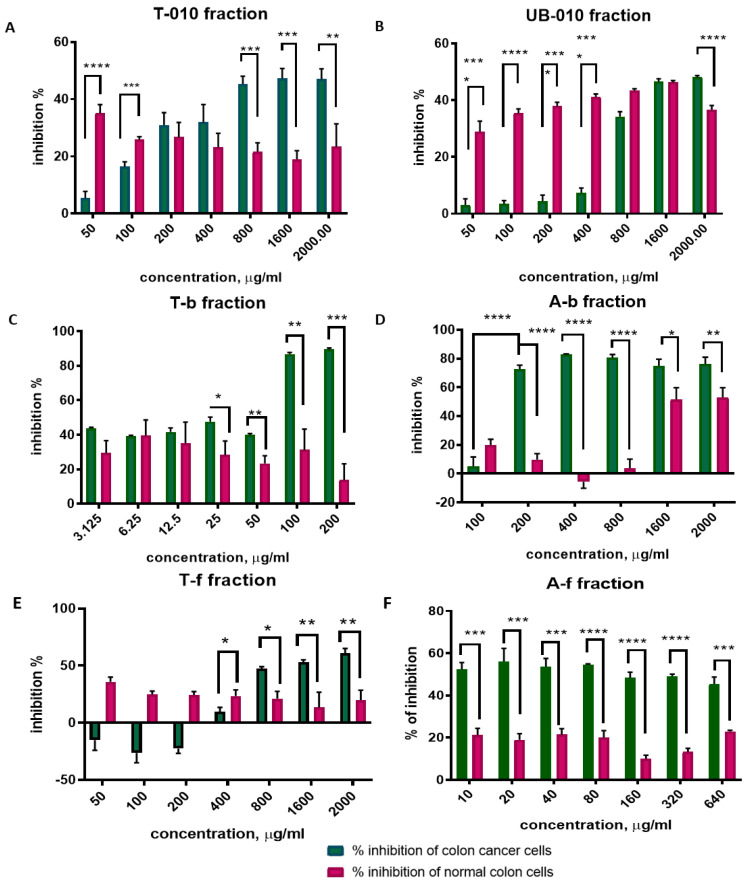
Inhibitory activity of PS fractions on the normal colon cell line CCD118 CO, compared with inhibition of colon cancer cells of the HCT−116 line. T−010 fraction (**A**), UB−fraction (**B**), T−b fraction (**C**), A−b fraction (**D**), T−f fraction (**E**); A−f fraction (**F**); (**A**,**C**,**E**) T−fractions; (**D**,**F**) A−fractions; UB–unbound fraction (**** *p*  <  0.0001, *** *p * <  0.001, ** *p*  <  0.01 and * *p*  <  0.05 versus control).

To evaluate 50% inhibition concentration (IC_50_) for the A-b, T-b, and T-f fractions, linear interpolation of semi-logarithmic inhibition dose-response curves was used. For the A-b fraction, the IC_50_ was 160 µg/mL with R square 0,8. For the T-f and T-b fractions, the linear interpolation gave IC_50_ values of 1657 and 78 µg/mL, respectively (R square 0.7 and 0.9). For the A-f fraction, this method did not show any valid statistical significance (low R square), as this fraction gave a non-monotonic response, with concentrations from 10 to 320 causing 50% inhibition of proliferation with no statistical difference between these concentrations (51% ± 3.9). Therefore, we selected the lower concentration of 10 µg/mL as IC_50_. In the case of the T-010 and UB-010 fractions, 50% inhibition of cell proliferation was not reached no matter how much the dose used was increased. Therefore, we considered 1600 µg/mL, the lowest dose that reached the inhibition closest to 50% for both fractions, as IC_50_ (Figure 3, Table 2).

IC_50_ values were also determined for each PS fraction for the CCD-18CO normal colon cell line. All T-media WCCPS fractions inhibited the normal cells, but the effect was more notable at low concentrations and decreased with the higher concentrations. The T-010 fraction inhibited 35% of normal colon cells at a concentration of 50 µg/mL; however, higher concentrations (100–400 µg/mL) caused a slight decrease in inhibition to 25–27%. Higher concentrations (800–2000 µg/mL) remained at 20–22% inhibition, but this was not statistically significant (*p*-value 0.0772) (Figure 3A). The T-b fraction at low concentrations (3.125–12.5 µg/mL) caused 30–40% inhibition of normal colon cells; however, higher concentrations (25–100 µg/mL) caused 20–25% inhibition and at 200 µg/mL, and the inhibition was only 15%, though this was not statistically significant (*p*-value = 0.0837) (Figure 3C). Incubation of cells with the T-f fraction showed a significant decrease; 50 µg/mL caused 36% inhibition, whereas at 100–800 µg/mL inhibition it was 20–25%, and at 1600–2000 µg/mL inhibition it was 12–18% (*p*-value = 0.046) (Figure 3E). Because of this concentration contrary effect, we were not able to calculate IC_50_ for normal colon cells.

The selective antiproliferative effect of the WCCPS fractions was estimated by calculating the therapeutic index (TI) using CCD-18CO normal colon cells. TI refers to the ratio of the dose of a drug that causes adverse effects at an incidence/severity not compatible with the targeted indication (Table 2). The UB fraction caused 30–47% inhibition of CCD-18CO normal colon cells, from the lower concentration of 50 to 1600 µg/mL respectively, with a slight decrease in inhibition at 2000 µg/mL. Therefore, the IC_50_ for this fraction was estimated at 800 µg/mL (Figure 3B).

Fractions from the A media were less toxic for normal colon cells in low concentrations compared to the T-media fractions. The IC_50_ for these fractions was estimated using a graph prism program. Therefore, the IC_50_ for inhibition of CCD-118CO cells by the A-b fraction was 1480 µg/mL, and the A-f fraction at 700 µg/mL (Figure 3D,F).

The TI was highest for the A-f fraction and high for the A-b fraction (70 and 9.25 respectively). These fractions were effective on cancer cells in lower concentrations and had a lower toxic effect on normal cells than the T-010, T-b, T-f, and UB-010 fractions.

The T media fractions gave high IC_50_ values for the colon cancer cells, but it was not possible to calculate IC_50_ values for normal colon cells. Consequently, no TI was determined for these fractions. However, for all these fractions, higher concentrations decreased their toxicity to normal cells (Figure 3). Therefore, as IC_50_ was not achieved, these fractions were less toxic for normal cells, and have the capability to have considerable TI. For example, the T-b fraction caused 40–50% inhibition of colon cancer cells at 3 to 75 µg/mL, but 86% inhibition at 100 µg/mL. At the same time, the T-b fraction inhibited normal cells by up to 40% at low concentrations, but at 100 µg/mL caused 30% inhibition and at 200 µg/mL caused 20% inhibition. Thus, for T-media fractions, instead of calculating TI, we presented a ratio of the percentage of inhibited cancer cells at the IC_50_ level, divided by the percentage of inhibited normal colon cells at the same concentration. Hence, this ratio indicated that at the IC_50_ levels, T-010 (1600 µg/mL) and T-f (800 µg/mL) fractions were 2.35 times more toxic for cancer cells than for normal cells, and the T-b fraction at 100 µg/mL was 2.86 times more toxic for cancer cells than for normal cells (Figure 3, Table 3).

These results confirm the potent anti-cancer effects of WCCPSs and also the selective nature of this anti-cancer activity. Among the 6 fractions, the A-fractions showed significant therapeutic indexes, i.e., high selectivity. Meanwhile, the application of T-fractions in the above-mentioned concentration range, in turn, did not allow us to determine IC_50_, but showed diminishing toxicity (in T-f fraction, significant reduction) and 2.3–2.8-fold decreased toxicity to normal cells. The A-b fraction showed an exponential escalation of IC_50_ and the A-f fraction showed its capacity to inhibit growth in around 50% of cells.

In order to analyze the mechanism of action of polysaccharides in tumor cells, we investigated WCCPSs effect on the HCT-116 cell cycle, morphology, and cell death.

### 3.3. Induction of Apoptosis and Cell Cycle Arrest

**Figure 4:**
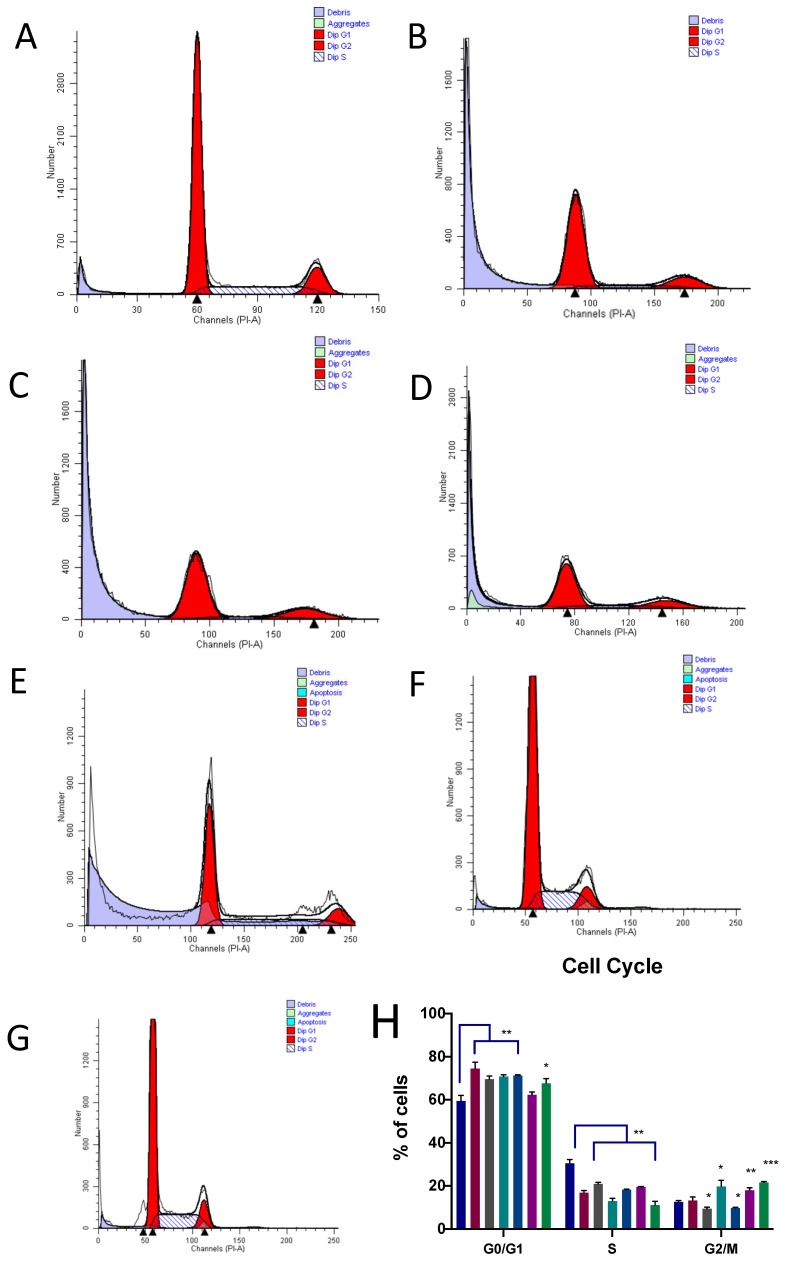

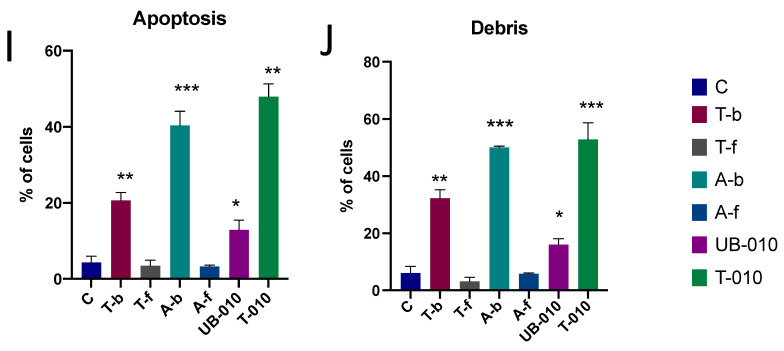
Cell cycle and apoptosis of HCT-116 colon cancer cells treated by WCCPS. (**A**)—untreated cells, (**B**)—A−b fraction, (**C**)–T−010 fraction, (**D**)—UB−010 fraction, (**E**)—T−b fraction (2× IC_50_), (**F**)—A−f fraction, (**G**)—T−f fraction, (**H**)—cell cycle, (**I**)—debris, (**J**)—apoptosis. (*** *p*  <  0.001, ** *p*  <  0.01 and * *p*  <  0.05 versus control).

We excluded the B-010 fraction from further investigations because it induced the proliferation of HCT-116 cancer cells. We tested the other fractions for their ability to induce apoptosis and cell cycle arrest because they caused inhibition of cancer cell growth and were less toxic to normal colon cells. Flow cytometry results revealed that treatment of HCT-116 colon cancer cells for 48 h with WCCPS fractions resulted in an increased proportion of cells at the G0/G1 phase and a significant decrease in the percentage of cells in the S phase (Figure 4).

In fact, the proportion of untreated control cells in the G0/G1 phase was around 59.6 ± 2.4%, whereas for treated cells the proportion ranged from 67.6 ± 2.2 to 74.5 ± 2.9%. The T-b fraction increased the proportion of cells in the G0/G1 phase up to 74.5 ± 2.4%, with the T-f fraction up to 69.6 ± 1.4%, the A-b fraction up to 69.3 ± 1.4%, the A-f fraction up to 71 ± 1.7% and the T-010 up to 67.6 ± 2.2%. There was also a decrease in the percentage of cells incubated with the UB-010 fraction in the S phase and an increase in G2/M compared to the control.

The percentage of cells in the S phase was around 30% (30.5 ± 1.8) in control cells, whereas for treated cells the proportion ranged from 11.2 ± 1.7 to 21 ± 0.7% (Figure 4H). All fractions caused a significant decrease of cells in the S phase. The T-b fraction decreased cells in the S phase to 17 ± 0.9%, the T-f to 21 ± 0.7%, the A-b to 13 ± 1.2%, the A-f to 17.9 ± 0.85%, the UB-010 to 19.6 ± 0.2%, and the T-010 to 11 ± 1.7%.

Our investigation revealed that some WCCPS fractions induced apoptosis (Figure 4I). Flow cytometry showed that apoptotic SubG1-phase cells were increased for T-010, UB-010, A-b, and T-b fractions. In particular, the highest apoptosis level (48% ± 3.4) was caused by the T-010 fraction, in comparison to the control (4.6 ± 1.6%). The A-b fraction showed a powerful effect as it caused programmed cell death in 40.3 ± 3.7% of cancer cells. The T-b and UB-010 fractions showed a moderate increase in cells in the SubG1 phase, at 20.6 ± 2% and 12.9 ± 2.5%, respectively. The percentage of cell debris was also high in the above-mentioned fractions and varied from 20 to 60% depending on the fraction (Figure 4J). However, no significant change in SubG1 or the amount of debris were observed after treatment with the A-f and T-f fractions, compared to the control (Figure 4I,J).

### 3.4. Electron Microscopy of HCT-116 Cells under the Influence of WCCPSs

#### 3.4.1. Transmission Electron Microscopy (TEM)

**Figure 5:**
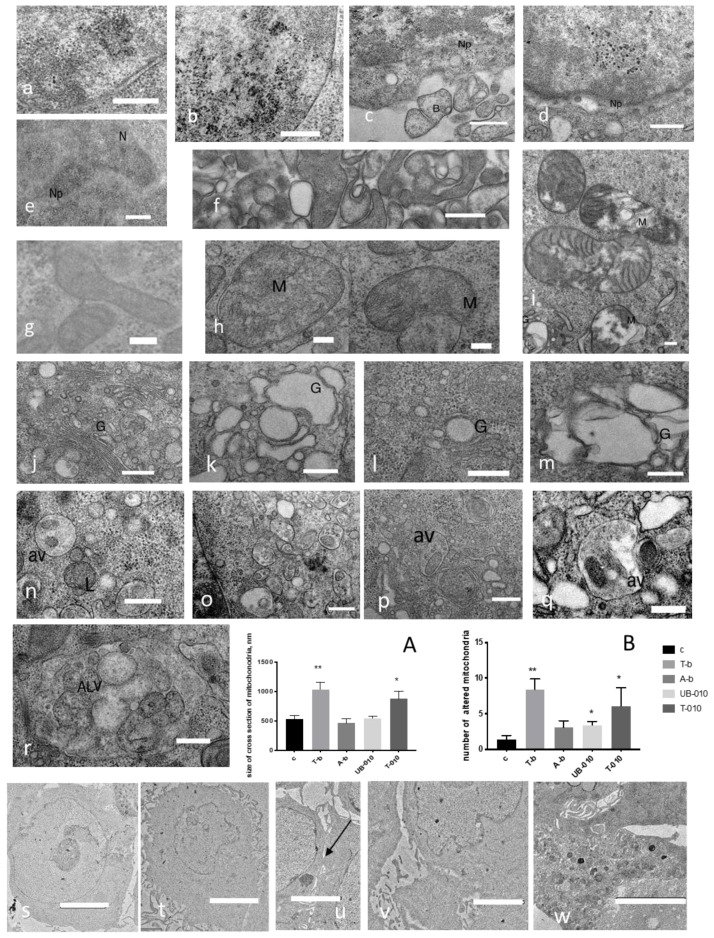
Transmission electron microscopy of the HCT-116 cell line treated by WCCPS in concentrations of IC_50_. (Scale bar: (**a**,**b**,**e**,**j**,**k**,**l**–**r**)—400, (**c**,**d**,**f**)—600, (**g**–**i**)—200, (**s**–**w**)—5000 nm), (**a**)—nucleus of untreated cells, (**b**,**c**)—nucleus with condensed chromatin and nuclear pores, apoptotic blebs of cells treated by T-010, (**d**)—nucleus of cells treated by T-b fraction, condensation chromatin, nuclear pores (Np) (2× IC_50_), (**e**)—nucleus of cells treated by A-b fraction, (**f**)—blebs in intercellular space between cells treated by UB-010 fraction, (**g**)—mitochondria of non-treated cells, (**h**)—mitochondria treated by T-010, (**i**)—mitochondria treated by T-b fraction, (**j**)—Golgi apparatus of non-treated cell, (**k**)—swollen Golgi apparatus in cells treated by T-010, (**l**) –Goldi (A-b fraction), (**m**)—Golgi apparatus (T-b fraction), (**n**)—lysosomes and autophagic vacuole of untreated cell, (**o**)—accumulation of multiple autophagic vacuoles with double and single membrane (T-010), (**p**)—autophagic vacuoles with double and triple membrane, (**q**)—vesicles in cell treated by T-b fraction, (**r**)—double membrane autophagic large vesicle in cell treated by UB-010 fraciton, (**s**)—untreated cell, (**t**)—cells treated by A-f fraction—brush borders, (**u**)—tight junctions between cells (A-f fraction), (**v**)—cryptae structures (A-f fraction), (**w**)—cells treated by T-f fraction—plenty of vesicles in intercellular space; (**A**)—the diameter of cross section of mitochondria, (**B**)—number of damaged mitochondria per picture. N-nucleus, Np—nuclear pores, M—mitochondria, G—Golgi apparatus, L—lysosome, av—autophagic vesicles; ALV—autophagic large vesicle, arrow—tight cell junctions. (** *p * <  0.01 and * *p * <  0.05 versus control).

TEM revealed changes in the nucleus, cytoplasm, and intercellular space of HCT-116 colon cancer cells under the influence of different wheat PSs (Figure 5).

Nucleus, blebs, and cytoplasm membrane. Compared to untreated cells (Figure 5a), treating colon cancer cells with the T-010 fraction led to the condensation of chromatin, the absence of the nuclear envelope, and blebs beside the cell (Figure 5b,c). Incubation with T-b and A-b fractions revealed fragmented chromatin very close to the nuclear envelope and visible nuclear pores (Figure 5d,e). The UB-010 fraction caused similar changes in the nucleus, but a more intensive release of blebs into intercellular space (Figure 5f). The A-f fraction caused the appearance of brush-like borders (Figure 5t) and tight junctions (Figure 5u) and cryptae structures (Figure 5v) between cells, compared to the control.

Mitochondria. Some WCCPS fractions caused significant effects on mitochondria such as damage and/or enlargement. The T-010 fraction (Figure 5g) caused swollen enlarged mitochondria, which were observed with disorganization of the inner membrane and partial damage, and the formation of almost empty white vesicles with barely visible thin mitochondria cristae compared to the control cells (h). Alterations in mitochondria were revealed after treatment of colon cancer cells with the T-b fraction (i): a doubly enlarged size of the mitochondria (1000 nm) in the cross-section compared to the control (500 nm) (Figure 5A) and an increased number of damaged and ruptured mitochondria (Figure 5B). There was no noticeable change in mitochondria size or damage upon treatment with the A-b fraction. The UB-010 fraction had a moderate increase in the number of damaged mitochondria, but no change in their size was observed. The A-f and T-f fractions did not show any changes in the size and consistency of mitochondria.

Golgi apparatus. In comparison to untreated cells (Figure 5j), the T-010 fraction caused swollen Golgi apparatus (Figure 5k). The A-b fraction also caused a moderate increase in the size of the Golgi apparatus (l). Incubation of HCT-116 cells with the T-b fraction revealed swollen enlarged and degraded Golgi (m). There was no visible evidence for changes in the Golgi apparatus under the influence of the UB-010 fraction.

Other changes in cytoplasm. Incubation with the T-010 fraction, unlike in control cells (Figure 5n), caused multiple autophagic vacuoles or apoptotic bodies (400 × 400 nm and less) with inclusions (or small particles) of different shapes (Figure 5o). The A-b fraction triggered the appearance of multilamellar bodies and autophagic vacuoles with a triple membrane with inclusions (600 × 400 nm and less) (Figure 5p). The T-b fraction differed by causing white empty vesicles in the cytoplasm in addition to vesicles with inclusions (700 × 500 nm) or autophagosomes/autolysosomes (Figure 5q). The UB-010 fraction caused the formation of large autophagic vesicles with a double membrane (2000 × 800 nm), containing multiple organelles or vesicles. Treatment of cells with T-f fraction allowed to observe formation of vesicles in cytoplasm and output of vesicles after cell degradation (Figure 5w).

Overall, we noted that the T-010, T-b, A-b, and UB-010 fractions caused significant changes in cancer cells: damaging the mitochondria, condensing chromatin, eliminating the nuclear envelope, producing a variety of vesicles inside and outside (blebs) the cell membrane, and multilamellar bodies. Interestingly, a variety of different sizes of autophagic vacuoles in the cytoplasm had inclusions such as granules and parts of degrading organelles.

#### 3.4.2. Scanning Electron Microscopy (SEM)

**Figure 6:**
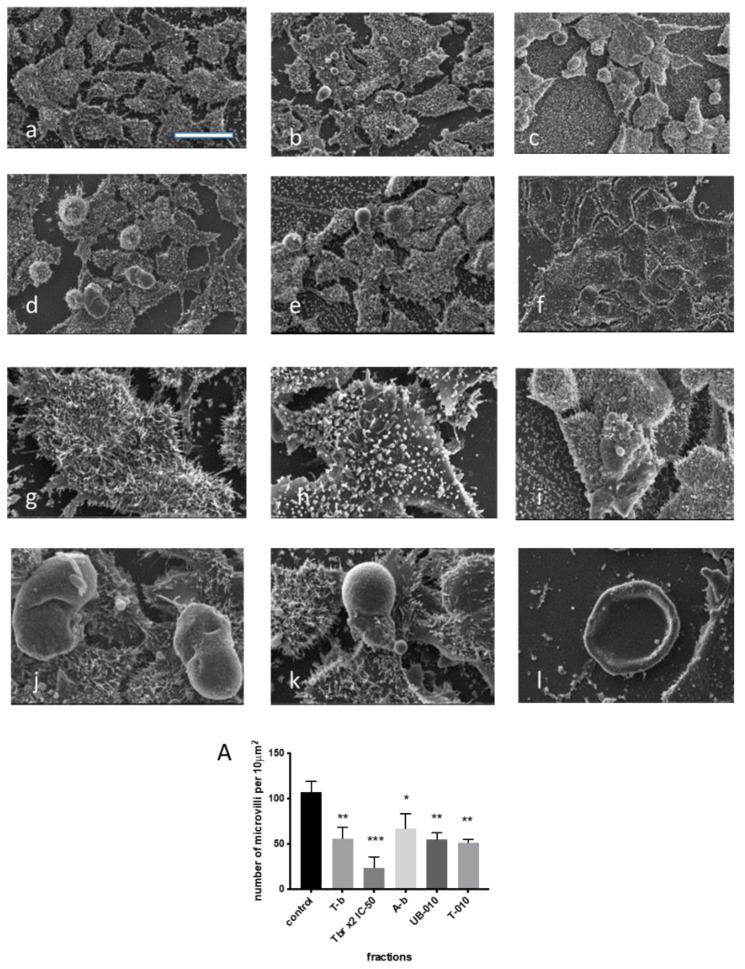
Scanning electron microscopy of HCT-116 cells treated by PS fractions. Magnification ×3000, 50 µm: (**a**)—untreated cells, (**b**)—T-010 fraction, (**c**)—A-b fraction, (**d**)—UB-010 fraction, (**e**)—T-b fraction IC_50_, (**f**)—T-b fraction with 2× IC_50_; Magnification ×9975, 10 µm: (**g**)—untreated cell, (**h**)—T-010 fraction, (**i**)—A-b fraction, (**j**)—UB-D fraction, (**k**)—T-b in concentration of IC_50_, (**l**)—T-b fraction 2 IC_50_, (**A**)—number of microvilli per 10 µm. (*** *p*  <  0.001, ** *p*  <  0.01 and * *p*  <  0.05 versus control).

Noteworthy changes in the morphology of HCT-116 colon cancer cells incubated with the T-010, A-b, T-b, and UB-010 fractions were also visible using SEM. We observed that the T-010 fraction, compared to control cells (Figure 6a,b), decreased the number of cancer cells, caused cell flattening, reduced the frequency and length of microvilli (Figure 6A), and stimulated the appearance of apoptotic bodies (or ball-like swelling of cells with the loss of microvilli) (Figure 6c,d). The A-b fraction also changed the morphology of cancer cells similar to the T-010 fraction, but with a ball-like swelling of cells and with no loss of microvilli (Figure 6e,f). In addition to similar changes, the UB-010 fraction contributed to the formation of two kinds of apoptosis bodies—some with remaining microvilli and some with the absence of microvilli (Figure 6g,h). Treatment of colon cancer cells with the T-b fraction in IC_50_ triggered the appearance of apoptotic bodies (Figure 6i,j), but in high concentrations (2× IC_50_) caused the drying of cells with only the membrane remaining (Figure 6k,l).

### 3.5. Mechanism of Anticancerous Action of WCCPS Fractions

**Figure 7:**
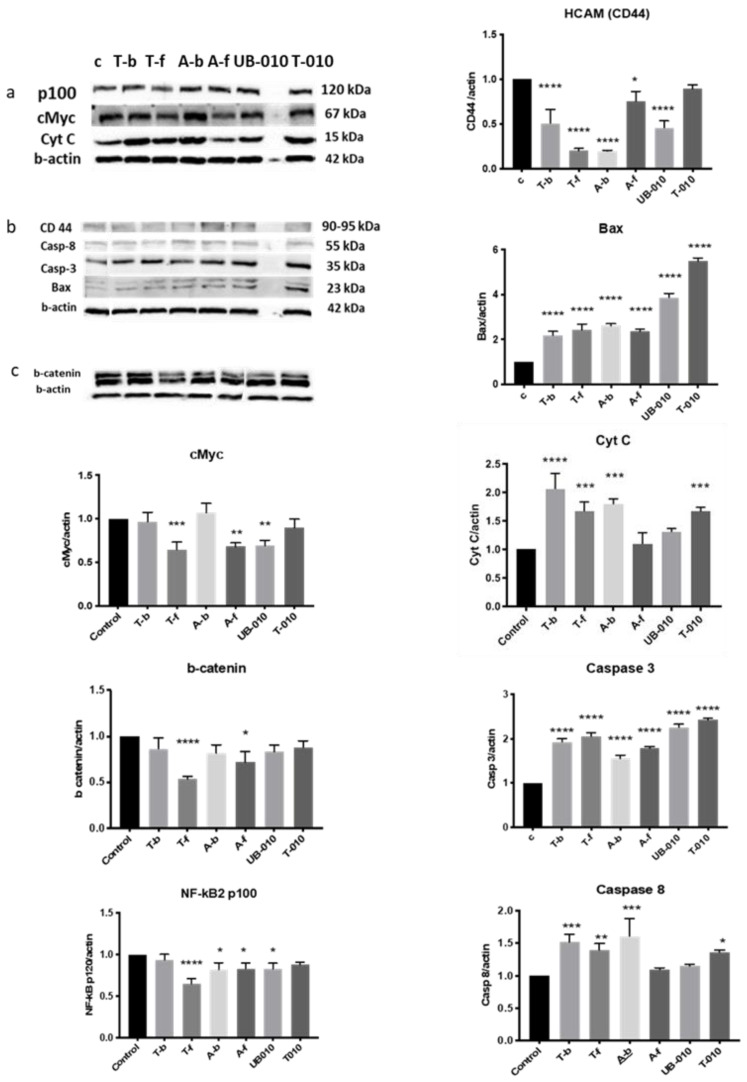
Western blot of different proteins related to cancer cell proliferation after treatment with PS fractions. Western blot quantification was normalized against the β-actin signal. Data were obtained from three independent experiments performed in duplicate and are expressed as mean  ± SD from three independent experiments performed in triplicate (**** *p*  <  0.0001, *** *p*  <  0.001, ** *p*  <  0.01 and * *p*  <  0.05 versus control). (**a**) membrane that contain proteins of NFkB p100, c-Myc, Cyt C; (**b**) membrane with CD-44, Caspases 3 and 8, and Bax, (**c**) membrane with beta-catenin.

Western blotting assessed the levels of proteins that are known to be involved in cancer cell death after treatment with the WCCPS fractions (Figure 7). The results of the experiment presented evidence that WCCPSs take part in important cellular signaling processes, altering key proteins of apoptosis, endothelial mesenchymal transition (EMT), and differentiation.

#### 3.5.1. c-Myc

The levels of c-Myc were compared to the relative value of the control (1). The T-f, A-f, and UB-010 fractions significantly decreased the levels of this protein (values are 0.6, 0.7, and 0.7, respectively (Figure 7)). Other fractions did not show any effect on c-Myc levels. It is important to point out that WCCPSs from long-term cultivation (T-f and A-f) and the neutral UB-010 fraction had the capability to inhibit c-Myc protein, suggesting reduced proliferation and increased differentiation.

#### 3.5.2. Beta-Catenin

Two fractions (T-f and A-f) reduced the levels of beta-catenin. Compared to the control value (1), the T-f fraction reduced levels by a value of 0.5, and the A-f fraction by a value of 0.7. No changes were caused by other fractions (Figure 7). In concordance with c-Myc reduction, the T-f and A-f fractions decreased beta-catenin levels, suggesting a synergetic effect and a path to reduce cell proliferation. In addition, beta-catenin plays a key role in EMT and cyclin D, an orchestrating protein of the cell cycle.

#### 3.5.3. NF-kB2

The levels of NF-kB p100 were reduced by the T-f, A-b, A-f, and UB-010 fractions. The T-f fraction reduced levels to 0.65 of control, whereas the A-b, A-f and UB-010 fractions reduced levels to 0.8. The T-010 and T-b fractions had no significant effect on the levels of Nf-kB2 p100 (Figure 7). The T-f, A-f, and UB-010 fractions showed their capacity, similar to c-Myc inhibition, to have a decreasing effect on the Nf-kB pathway, participating in differentiation processes.

#### 3.5.4. HCAM (CD44)

All fractions, except T-010, reduced the levels of HCAM (CD44). The T-b and UB-010 fractions reduced the levels to 0.5, relative to the control (1). The T-f and A-b fractions reduced levels down to 0.2. The A-f fraction reduced levels to 0.7 relative to the control (Figure 7). These fractions showed their activity in promoting the differentiation of colon cancer cells, especially the T-f and A-f fractions.

#### 3.5.5. Bax

The levels of Bax were increased by all WCCPS fractions. The levels were doubled (relative value of 2) when treated with T-b, T-f, A-b, and A-f fractions compared to the control (1). However, the UB-010 and T-010 fractions increased Bax levels by 3.5 and 5.2-fold, respectively (Figure 7). Thus, the T-010 and UB-010 short-term cultivated fractions have a high capacity to induce the release of Bax, a protein known to trigger an apoptotic signal and promote cytochrome c release.

#### 3.5.6. Cytochrome c

Cytochrome c levels were increased by the T-b, T-f, A-b, and T-010 fractions. The levels were doubled by the T-b fraction (2), increased 1.6-fold by the T-f fraction, 1.7-fold by the A-b fraction, and 1.6-fold by the T-010 fraction. No significant changes were observed for the A-f and UB-010 fractions (Figure 7). Therefore, most fractions participated in a process of apoptosis by stimulating cytochrome c release; however, the A-f fraction did not show any change in the levels of this protein.

#### 3.5.7. Caspase 3

The levels of pro-caspase 3 were increased by all fractions. The increases were 1.9-, 2.0-, 1.5-, 1.7-, 1.15-, and 1.25-fold, respectively, for fractions T-b, T-f, A-b, A-f, UB-010, and T-010 (Figure 7). Therefore, all WCCPS fractions increased caspase 3 levels, a key mediator not only for apoptosis but also for other processes such as proliferation and differentiation.

#### 3.5.8. Caspase 8

Four fractions, namely T-b, T-f, A-b, and T-010, increased levels of caspase 8 by 1.5-, 1.4-, 1.57-, and 1.3-fold, respectively. The UB-010 and A-f fractions did not cause any increase in caspase 8 (Figure 7). These findings suggest that all T-fractions and the A-b fraction are involved in an extrinsic apoptotic pathway, confirming the capability of these fractions to induce apoptosis in vitro.

Thus, all fractions caused significant increases in the levels of Bax and caspase 3. The T-b, T-f, A-b, and T-010 fractions also caused a significant escalation in cytochrome c and caspase 8 levels. The T-f, UB-010, and A-f fractions decreased levels of c-Myc. The levels of b-catenin protein were inhibited by the T-f and A-f fractions. Treating cells with the AB-f, T-f, UB-010, and A-b fractions led to a reduction in NFkB p100 levels. A notable decrease in CD44 protein levels was stimulated by all fractions.

## 4. Discussion

The search for new molecules for uses as possible anticancer agents has contributed to the investigation of the anticancer activity of polysaccharides (PSs) from various sources [50,51]. In this study, we investigated polysaccharides obtained from suspension cultures of wheat, referring to the type of media and culture time. WCCPS fractions showed different levels of anti-cancer activity, and therefore different doses of tested PS fractions were necessary to achieve a direct inhibitory effect on cancer cells. WCCPSs also have different cytotoxic effect on normal cells: The A-fractions were less toxic for normal cells in a dose-dependent manner, however, the T-fractions showed reverse dose-response dependency, where lower concentrations inhibited more than higher concentrations. This phenomenon was found only with the T-fractions and only with the normal colon cell line CCD-18CO. An important note is that the A-f fraction had a similar response (around 50% of inhibition) over a wide range of concentrations. There is some evidence that certain substances may have higher toxicity in lower doses than in higher doses, or in some other unusual dose–response relationship. Other researchers have reported this kind of “non-monotonic” cell response for endocrine disrupters, capable of interacting with cellular hormone receptors. For example, atrazine, biphenol A (BPA), and vinclozolin have been shown to have an unusual dose–response dependency [52]. It was proposed that a non-monotonic response could be explained by acting at multiple molecular targets of different affinity, by an antagonistic effect, negative regulation, or receptor desensitization, etc. [53]. It is suggested in the literature that there is a need for further research and identification of substances with non-monotonic dose–response, because they are rarely published [54]. However, no previous evidence suggests this reverse dose-dependency for polysaccharides.

In our study, we were able to identify distinctions in mechanisms of action and morphological changes at a cellular level in WCCPS fractions with differences in media and the cultivation time of wheat cells. Many investigations of natural polysaccharides from herbs, intact plant parts, fungi, or bacteria have been conducted. In these studies, the authors indicated that different concentrations are necessary to achieve the IC_50_ for cancer cells: Studies frequently suggested that 30% inhibition or IC_30_ was the maximum, as it is difficult to achieve 50% mortality for cancer cells using a natural PS [7,55]. In our study, different IC_50_ levels were achieved for most of the tested PSs. Thus, a relatively low level of IC_50_ of HCT-116 colon cancer cells was achieved using the A-f fraction—10 µg/mL, the A-b fraction -160 µg/mL, and the T-b fraction—50–100 µg/mL. The T-010, UB-010, and T-f fractions exhibited a much higher IC_50_ (1600 µg/mL). Thus, our research showed that, overall, the A-fractions have considerable therapeutic indexes, and were less toxic for normal cells. The T-fractions were revealed to be less toxic for normal cells in the concentrations inhibiting 50% or more of colon cancer cells. This is interesting because previous work [33] suggested that polysaccharides from ABA treated wheat cells (specifically the A-b fraction) were quite different from those from 2,4-D treated cells (specifically the T-010-fraction). Indeed, the A-b fraction was enriched in glucose, probably from β-glucans, compared to the T-010 fraction which had a monosaccharide composition suggestive of a more equal mixture of arabinogalactans, glucuronarabinoxylans, β-glucans, and xyloglucans (see Supplementary Data, Figure S1A). The fractionation of the T-010 fraction into bound and unbound fractions by ion exchange also changed the bioactivity profile, with the bound fraction actually stimulating colon cancer proliferation. This was also intriguing as the bound fraction contained the acidic arabinogalactans and glucuronarabinoxylans with the unbound fraction containing the neutral glucans and xyloglucans (see Supplementary Data, Figure S1B). It was intriguing that the T-f fraction also stimulated proliferation but only at low levels (Figure 3), as this fraction had the highest levels of arabinose after the bound fraction, again suggesting that arabinogalactans and glucuronarabinoxylans may be involved in this stimulation. In fact, as well as the lower glucose and higher levels of rhamnose/arabinose, and galactose in the T-type fractions over the A-fractions, there was also a trend to greater diversity in monosaccharide composition (and predicted polysaccharide diversity) in the PS samples from the older wheat cultures (e.g., A-f and T-f). Overall, the ratio of monosaccharide composition showed that the absolute content of Glc and the ratio of Glc:Ara:Man appeared to be the main factor for the structure-functional relationship and determining anticancer activity. For example, the fractions A-b and T-b with high anticancer effects have Glc:Ara:Man ratios of 1:92:3 and 8:57:4, respectively.

Indeed, some studies have discussed that the length of cultivation of a plant cell culture could alter the output chemicals in the media [56]. For instance, a medium of rice cell suspension culture had a considerable antitumor effect after 3 weeks of cultivation, which was suggested to be due to the appearance of secondary metabolites during longer-term cultivation [57]. The precise mechanism is unclear but needs to be investigated with future research. However, it could be related to an elicitor influence (as biotic stress) to produce more secondary metabolites. Polysaccharides are reported to be possible elicitors [58,59,60], that were added to media to increase and accelerate the output of metabolites. In our case, PSs produced by wheat callus cells during cultivation with phytohormones, and, perhaps, accumulating and transforming in the cell culture media during long-term cultivation, stimulated the production of secondary metabolites.

Our results showed that some WCCPSs have an effect on colon cancer cells in relatively lower or similar concentrations than have been found in previous studies on different cancer cell lines, including colon cancer [61,62,63,64,65,66,67,68]. For instance, Cheng et al. showed that Ginseng PS (GPS) caused inhibition of human colon cancer HT-29 cells at 600 μg/mL [69], Ma and colleagues provided evidence that PS extracted from hawthorn (*Crataegus*.) effectively inhibited cell growth (up to 74%) in a concentration range of 500–1000 µg/mL [70]. Moreover, flow cytometry revealed that treatment with all fractions of WCCPSs enabled cell cycle arrest at the G0/G1 phase. In addition, the A-f and T-f fractions could decrease the percentage of cells in the G2/M phase, while the A-b, UB-010, and T-010 fractions could increase the proportion of cells in this phase. The result was partially consistent with some other studies on plant polysaccharides. For example, it was shown that cell cycle arrest could take place during the G0/G1 and S phases. In contrast with our findings, Liang (2014) et al. [71] showed that incubation of HCT-116 cells with much higher concentrations (1.25, 2.5, 5, and 10 mg/mL) of GLP significantly increased the S phase up to 19.26%. Our results showed, in contrast, a significant decrease in the S-phase. Meanwhile, Zhang found that PS from Peony Seed Dreg CASS induced accumulation of all the cells in the G0/G1 phase in Hela cell lines, except for MCF-7 cells which were arrested in the S phase [72].

It is well known that apoptosis (type I programmed cell death) is an essential mechanism in maintaining the balance between cell death and proliferation. Its deregulation leads to cancer cell progression. Notably, many cancer cells appeared to resist apoptosis signaling, suggesting the search for compounds that induce apoptosis of cancer cells [73,74]. In this context, polysaccharides have been described as natural compounds with anticancer activity mediated by apoptosis. Hence, Lin et al. provided evidence that the *Scleromitrion diffusum* polysaccharide (SDP) in high doses inhibited tumor growth by apoptosis such as cisplastin-positive drugs [75]. In addition, Li et al. found that *Polygonatum cyrtonema Hua* polysaccharide induced Hela cell inhibition of proliferation by regulating the cell cycle and different apoptosis pathway-related genes [76]. In our attempt to understand the mechanism by which WCCPSs induce inhibition of growth, we performed an apoptosis assay. Our results demonstrated that the A-f and T-f fractions, which were obtained after long-term cultivation, had no changes in either apoptosis level or cell debris compared to the control. However, some fractions of WCCPSs induced apoptosis. After 48 h of WCCPS exposure, 1600 µg/mL of T-010 fraction caused apoptosis of 48% of cells, 160 µg/mL of A-b fraction—40%, 1600 µg/mL of UB-010 fraction—13.5%, 100 µg/mL of T-b fraction—16.7% while control cells showed only 4.5% of apoptosis. Debris from other fractions varied from 20 to 60% compared to the control (10%). This level of apoptosis triggered by WCCPSs is considerable, compared to some other plant derived polysaccharides. For instance, GLP polysaccharides induced 10–15% of apoptosis of the HCT-116 cell line in much higher concentrations, 1250–10,000 µg/mL during 24 h cultivation [62]. However, polysaccharides from red algae (ASPE) had a reported effect of low concentrations (50 µg/mL) causing an apoptosis of 50% of cells [77]. Polysaccharides from *Larimichthys crocea* swim bladder induced 37.2% apoptosis of HCT-116 cancer cell line in a concentration of 400 µg/mL [78].

Apoptosis was also confirmed by transmission and scanning electron microscopy for fractions with high apoptosis levels—namely T-010, UB-010, A-b, and T-b. The following signs of apoptosis were present in our study: defragmentation and condensation of chromatin [79,80]; damaged and enlarged mitochondria with disorganized inner membranes; degrading mitochondria; the appearance of plenty of autophagic vacuoles with particles inside; swollen Golgi apparatus; absence of the nuclear envelope; blebs and multilamellar bodies. According to TEM observation, the accumulation of autophagic vacuoles with one, double and triple membranes, could signify the autophagic flux block, which means that the cell is dying. Several works indicated that the accumulation of autophagosomes played a role in the induction of cell death by the blockage of autophagic flux [81]. Notably, the size of mitochondria was significantly changed by the T-b fraction, and the number of damaged mitochondria with absent or damaged cristae was increased by 3 fractions (T-010, UB-010, and T-b). SEM revealed apoptotic bodies and a diminishing number of microvilli under the influence of WCCPSs.

As A-f and T-f fractions showed no signs of apoptosis, but reduced the S phase of the cell cycle, it is expected that these 2 fractions took part in other processes, related to the inhibition of proliferation. In TEM, it was shown that the A-f fraction may have had an impact on differentiation, as we observed brush borders, tight junctions between cells, cryptae structures [82], which are considered markers of a differentiated cell. In addition, the T-f fraction induced the appearance of vesicles of mucus in the cytoplasm and their subsequent release into the extracellular space, which were characteristics of the differentiation [83,84].

There were two pathways for triggering apoptosis: intrinsic or mitochondrial, and extrinsic, through death receptor. Both pathways led to the activation of caspases, and triggered intracellular changes such as chromatic condensation, DNA fragmentation, membrane blebbing, and cell shrinkage [85]. There were plenty of signaling pathways through which apoptosis could occur, involving p53, Bcl-2, NFkB, MAPK, and other related proteins [86,87]. It was reported that the cytotoxic effect of plant polysaccharides has been achieved through one or more mechanisms (MAPK, NF-kB, JNK, p38, p53, PARP, etc.) [77,88]. In our study, we were able to reveal increased Bax, cytochrome c, and caspase 3 and 8 levels, which confirmed apoptosis in colon cancer cells caused by WCCPSs. This mechanism implied the activation of apoptosome-associated proteins, increased APAF-1, and caspases 3 and 9, the increased levels of which suggested longer patient survival with colorectal cancer [89,90,91,92,93,94]. All fractions stimulated the release of pro-apoptotic Bax protein, which implied that WCCPSs could target the Bcl-2 family, which triggered intrinsic pathways. Bax, in turn, played a key role in the release of cytochrome c from mitochondria to cytoplasm [95], which is also increased by WCCPS treatment (T-b, A-b, T-f, and UB-010). This cascade was followed by the activation of caspase 9 and the eventual activation of the terminal apoptosis executor—caspase 3, which was also increased by the influence of all WCCPS fractions. However, the A-f and UB-010 fractions have not shown an ability to induce significant cytochrome c release compared to the control but were capable of triggering significant Bax and caspase 3 expression. This could be explained by other compounds described in literature—SMAC/Diablo and HtrA2/Omi, that can be released separately from cytochrome c and cause activation of caspase 9 and caspase 3 [96,97]. Summarizing this evidence, we can conclude that T-010, UB-010, A-b, and T-b fractions possess the ability to promote the intrinsic mitochondrial pathway of apoptosis. However, T-f fractions have not shown the ability to cause apoptosis and the formation of debris in a cycle/apoptosis assay.

In addition, there was an extrinsic pathway which occurred through the activation of death receptors (Fas, TNFR-I, TRAIL, etc.). Activated death receptors initiated caspase 8 activation, followed by triggering caspase 3, which, in turn, executed apoptosis [98]. Four fractions (T-b, A-b, T-f, and T-010) were able to increase the level of caspase 8; therefore, they activated the extrinsic pathway of apoptosis, except for the T-f fraction, which did not trigger apoptosis according to the cell cycle assay.

It was also revealed that the T-f and A-f fractions inhibited the expression of the b-catenin protein, suggesting the blocking of the Cycline D1 protein and the APC (adenomatous polyposis coli) gene pathway, thus limiting cell proliferation [90,99]. It has been previously suggested that the decreased levels of b-catenin are associated with the increase of drug-initiated inhibition of cell growth [100]. These data were confirmed by decreased levels of c-Myc protein under the treatment with A-f, T-f and UB-010 fractions. It has been shown that overexpression of c-Myc protein is accompanied by increased b-catenin levels, that enhanced neoplastic cell proliferation [101]. Therefore, the A-f, T-f, and UB-010 fractions were capable of inhibiting HCT-116 colon cancer cell growth by targeting APC genes.

Since the T-f and A-f fractions did not express apoptosis or other cell death signs in apoptosis analysis (though they caused a cell cycle phase shift), it could be suggested that they participated in differentiation processes by decreasing c-Myc and beta-catenin levels. c-Myc protein overexpression promoted cell proliferation and oncogenesis, and in addition, it reduced the differentiation level of cells. Consequently, a decrease in c-Myc protein caused a shift for cancer cells to become more differentiated, capable of growing at much lower rates than cancer cells [102,103]. Moreover, the T-f and A-f fractions were shown to significantly decrease the level of CD44 expression, which also confirmed the ability of these fractions to promote differentiation in colon cancer cells. In addition, it is important to mention that in our cell cycle assay it was shown that only the T-f and A-f fractions are blocking cell cycle at all points: G0/G1, S, and G2/M. In general, cell cycle arrest contributes to differentiation, not being an ultimately necessary element of differentiation, but accompanying it by downregulating cyclins or by activating cyclin-dependent kinase (CDK) inhibitors [104].

The T-f fraction, moreover, expressed high cytochrome c levels, without causing apoptosis or other cell death, according to cell cycle and apoptosis analysis. This phenomenon can be explained by another quality of cytochrome c, which, besides causing apoptosis, can play a role in cell differentiation. This non-apoptotic increase in differentiation was usually also accompanied by the cleavage of caspases, without inducing cell death [105].

It was shown that only the T-f, A-f, A-b, and UB-010 fractions reduced levels in NFkB 2 p100 protein, which is the early form of active NFkB2 p52 protein [86]. In turn, the T-010 and UB-010 fractions induced inhibition of the p52 protein. It is known that there are two types of NFkB pathway, which are triggered differently and induce the activation of different genes: classical (NF-kB 1, throughout p65/p50 proteins) and alternative (NK-kB 2, throughout p100/p52 proteins) [106]. Both mechanisms are important in tumorigenesis; however, the alternative mechanism has not been deeply investigated in colon cancer cells. Moreover, there are some anticancer drugs that have been tested for downregulation of the classical NF-kB pathway, and fewer compounds that have been tested for the initiation of the suppression of an alternative pathway [107], which is a subject for new drug research and application [108]. Therefore, our results suggest that the A-b and UB-010 fractions can also induce apoptosis and the T-f, A-f, A-b, and UB-010 fractions trigger differentiation through the NFkB alternative pathway and could be considered for further testing as an anti-NFkB agent in colon cancer treatment.

Finally, we found that WCCPS fractions, except for T-010, also attenuate the expression of CD44 protein. It was reported that the CD44 receptor is related to colon cancer cell progression and has been reported to be a promising prognostic indicator for this pathology [109]. Notably, CD44 expression can be correlated with the Wnt pathway and epithelial–mesenchymal transition. Moreover, CD44 is a biomarker of cancer stem cells (CSC) in some tumors and is considered to contribute to cancer metastasis and growth [110]. It was shown that the differentiation of breast cancer stem cells occurred by the knockdown of CD44 and caused the loss of the CSC phenotype along with the reduced expression of Bcl-2 and Muc-1 proteins, reducing metastasis and tumorigenesis [111]. Therefore, the T-b, T-f, A-b, A-b, and UB-010 fractions could be considered capable of reducing the expression of CD44, impacting the differentiation and contributing to diminishing cancer progression and metastasis. Thus, we observed convincing evidence that some WCCPSs are able to induce differentiation of cancer cells. In the literature, we found several studies that demonstrated the ability of natural polysaccharides to stimulate differentiation. Chen and collaborators reported that PSs from the *Cordyceps sinensis* medicinal plant induced the differentiation of human leukemic U937 cells through increased secretion of cytokines from a monocyte culture pretreated by PSs [112]. PSs obtained from rice also had an effect on cancer cell differentiation through immune cell stimulation [113]. According to Hsu et al., PSs from *Ganoderma lucidum* fungus were shown to have a direct differentiation effect on human leukemia THP-1 cells and were proposed for possible use in differentiation therapy [114]. Recently, it was found that the *Angelica sinensis* polysaccharide could induce the erythroid differentiation of human chronic myelogenous leukemia k562 cells [115].

Our study allowed us to assume that different fractions, depending on cell growth media (A- and T-fractions) and on the cultivation time, showed different levels of cytotoxicity, and activated distinct processes and pathways. The A-fractions tended to be effective in killing cancer cells in lower concentrations and had low cytotoxicity towards normal colon cells. T-fractions have higher cancer cell inhibition doses, and higher toxicity for normal cells, compared to A-fractions. Therefore, A-fractions have considerable therapeutic indexes. In addition, after testing fractionated PSs, we concluded that fractionation is not advisable for obtaining a PS for tumor cell inhibition purposes, because acidic (B-010) fractions stimulate the proliferation of cancer cells, and basic (UB-010) fractions show higher toxicity toward normal colon cells.

PSs from short and medium-term cultivated cell cultures (T-010, UB-010, A-b, and T-b) induced intrinsic and extrinsic apoptosis pathways (except for UB-010). These PSs may also contribute to the differentiation of cancer cells according to the reduction of CD44 protein expression.

PS fractions which were obtained from long-term cultivated cultures (T-f and A-f) were shown to inhibit APC and NFkB pathways, and to reduce CD44 levels, suggesting that they act as differentiation agents.

## 5. Conclusions

Among the WCCPS compounds tested, six fractions were revealed to have an antiproliferative effect on the colon cancer cell line HCT-116. Fractionation by ion-exchange chromatography was not effective (specifically, resulting in bound fraction rich in arabinose and galactose) in obtaining PS with anticancer properties and that crude WCCPSs fractions were more effective in inhibiting cancer cell growth. This finding provides evidence that acidic PSs are not effective against cancer cells. A-fractions were active against cancer cells in relatively low concentrations and had considerable therapeutic indexes, providing evidence for their potency as possible antitumor agents. T-fractions were shown to inhibit cancer cells at higher concentrations, compared to A-fractions, and to have higher concentrations for inhibiting normal colon cells, but have been proved to be less toxic for normal cells than for cancer cells at IC_50_ level. In addition, there was a difference in the anti-cancer mechanism of action of WCCPS fractions, caused by differences in phytohormonal balance of the initial cell culture and cultivation time. Taken together, our results provide, for the first time, a simple and sustainable method to produce wheat cell culture polysaccharides with high anticancer effect achieved by cell cycle arrest, apoptosis and differentiation. The renewal potential of our cell cultivation system transforms it into a very economical tool with high production efficiency. More in vitro and in vivo studies need to be done to elucidate the mechanism of action of WCCPSs in normal cells and other types of tumors.

## 6. Patents

Patent: P202230419.

## Figures and Tables

**Table 1:** % Monosaccharide composition of WCCPSs fractions.

	T-010	B-010	UB-010	A-b	A-f	T-b	T-f
Ara	9.1	32.6	8.7	1.4	7.35	7.9	16.2
Gal	10.2	20.9	5.9	0.76	3.3	15.1	9.6
Xyl	6.7	18.3	9.7	1.9	4.8	13.86	13.6
Glc	73.9	3.3	75.6	91.4	83.2	56.6	58.0
GlcUA	0	24.9	0	0.4	1.1	1.74	2.2
GalUA	0	0	0	0.6	0.13	0.2	0.3
Man	0	0	0	3.5	0.02	4.6	0

**Table 2:** Estimated IC_50_ and therapeutic index for each polysaccharide fraction.

Fraction	HCT-116 IC_50_ (µg/mL)	CCD-18CO IC_50_ (µg/mL)	Therapeutic Index (TI)
T-010 **	1600	ND	NC *
B-010	NM *	2500	NC
UB-010 ***	1600	800	0.5
T-b	78	ND	NC
A-b	160	1487	9.25
T-f	1657	ND	NC
A-f	10	700	70

* Not Measurable: cell proliferation stimulated and IC_50_ could not be measured; NC −not calculable, ND—not determined because no higher concentrations applied; ** IC_50_ was considered at 47 ± 35% inhibition; *** IC_50_ was considered at 46.2 ± 2% inhibition.

**Table 3:** Ratio of the percentage of inhibited HCT-116 cells at a concentration that inhibits a number of cells closer to the IC_50_ level and a percentage of CCD-18CO cells at the same concentration for T-fractions.

Fraction	HCT-116 (%)	CCD-18CO (%)	Ratio
T-010 (1600 µg/mL)	47	20	2.35
T-b (100 µg/mL)	86	30	2.86
T-f (1600µg/mL)	50	20	2.50

## Data Availability

Not applicable.

## Supplementary Materials

The following supporting information can be downloaded at: https://www.mdpi.com/article/10.3390/pharmaceutics14051100/s1, Figure S1: Relative monosaccharide composition of polysaccharide fractions.

## Abbreviations

WCCPSs	Wheat cell culture polysaccharides
CRC	Colorectal cancer
APC	Adenomatous polyposis coli
EMT	Endothelial mesenchymal transition
