# Supplementary material for: Anti-Cancerous Potential of Polysaccharides Derived from Wheat Cell Culture
Source: Pharmaceutics. 2022 May 20;14(5):1100. doi: 10.3390/pharmaceutics14051100 (PMC9147229; doi:10.3390/pharmaceutics14051100)
Supplement: Supplementary file 1 [file pharmaceutics-14-01100-s001.zip › pharmaceutics-1676310-supplementary.pdf]

Supplementary Data

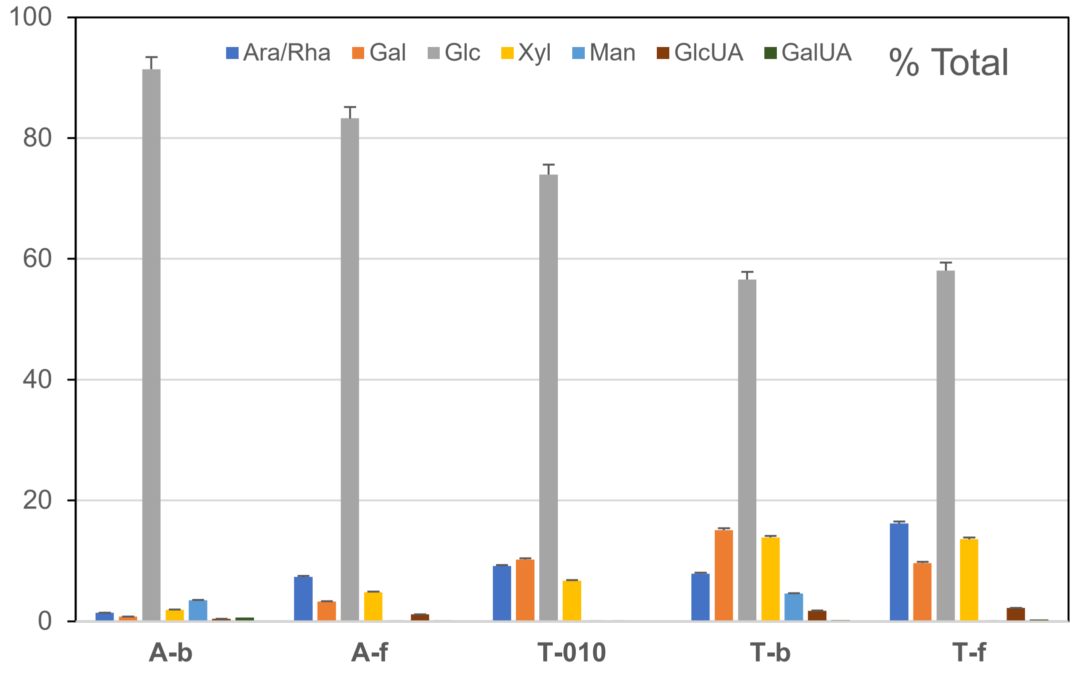

(**A**)

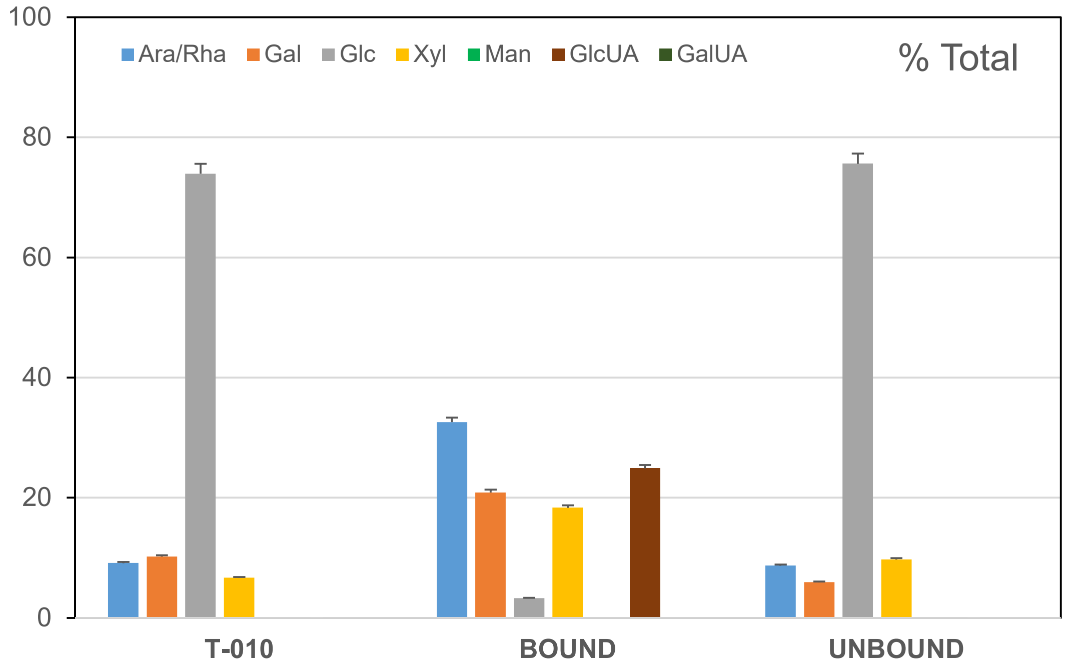

(**B**)

Figure S1. Relative monosaccharide composition of polysaccharide fractions determined by high performance anion exchange chromatography (HPAEC). (**A**) The main T and A fractions. (**B**) Fraction T-010 and the bound and unbound fractions.
